# Elimination or more accurate estimation? Investigation of trends in malaria diagnoses in the Ouest Department of Haiti from 2008 to 2017

**DOI:** 10.1371/journal.pone.0198070

**Published:** 2018-06-07

**Authors:** Thomas A. Weppelmann, Caroline J. Stephenson, Elisha Musih, John B. Dame, Marie Y. Remy, Robert Nicolas, Michael E. von Fricken

**Affiliations:** 1 Herbert Wertheim College of Medicine, Florida International University, Miami, FL, United States of America; 2 Department of Global and Community Health, George Mason University, Fairfax, VA, United States of America; 3 College of Veterinary Medicine, University of Florida, Gainesville, FL, United States of America; 4 African Methodist Episcopal Church Service and Development Agency, Inc., Washington, DC, United States of America; Quensland University of Technology, AUSTRALIA

## Abstract

**Background:**

According to the 2016 World Malaria Report, the malaria incidence in Haiti declined by > 40% between 2010 and 2015. Though elimination efforts have likely contributed, this time period also corresponded to a national change in diagnostic methods.

**Methods:**

Monthly reports of aggregated patient data were acquired from five clinics in the Ouest Department of Haiti. Generalized linear models were used to compare the number of febrile patients tested, the number of positive tests, and the proportion of tests that were positive (TRP) before and after the national adoption of rapid diagnostic tests (RDTs).

**Results:**

Prior to the earthquake when microcopy was used for diagnosis, a total of 1,727 patients with 557 (32.3%) positive; post-earthquake testing was reduced and the TPR was variable; during the post recovery period when RDTs were used exclusivly, a total of 5,132 patients were tested using RDTs, only 83 (1.62%) were positive. Compared to the pre-earthquake period, there was a 69% increase in the number of patients tested (IRR: 1.69; 95% CI IRR 1.59, 2.79), and a 97.0% decrease in the proportion of patients with a positive test result (IRR: 0.03; 95% CI IRR 0.02, 0.04) in the post-recovery period.

**Conclusions:**

While the decline in malaria indicators between 2010 and 2015 has been cited as evidence of progress towards elimination, these reports derived estimates of the malaria burden in Haiti using two different diagnostic tests. Thus, comparison of these periods in the context of malaria elimination should be made with caution.

## Background

Hispaniola, consisting of Haiti and the Dominican Republic, is the only remaining island in the Caribbean with endemic malaria, with more than 95% of all cases reported from Haiti [1]. In response, a bi-national plan between Haiti and the Dominican Republic was created to eliminate malaria by the year 2020 [2]. To reach this goal, Haiti has implemented several policies, including the recommendation to combine single dose primaquine with standard chloroquine therapy for confirmed malaria patients in order to block gametocyte transmission, efforts to strengthen reporting and surveillance, and a change in the diagnostic method from microscopy of blood smears to the use of rapid diagnostic tests (RDTs) [2]. Prior to 2012, malaria diagnosis in Haiti was exclusively based on results from parasitological microscopy of blood smears from suspected patients, which are highly dependent upon the type of equipment used and the level of training of the microscopist [3]. After the 2010 earthquake, the use of malaria RDTs was temporarily approved for 90 days [4], setting the groundwork for the permanent adoption of RDTs as the primary method of diagnosis in 2012. The Haitian government, in partnership with the United States Centers for Disease Control and Prevention (CDC), approved three brands of RDTs for use in Haiti. These brands exclusively target histidine-rich protein II antigen (HRP2) expressed by *Plasmodium falciparum* and were found to have sensitivity between 84–100% and specificity between 91–94% during in-country performance testing [5]. RDTs are now used countrywide for malaria diagnosis, which are reported to the MSPP (Ministère de la Santé Publique et de la Population) to derive estimates of the disease burden of malaria in Haiti [6].

This change in policy has corresponded with a large reduction in the number of confirmed malaria cases reported to the MSPP; with the 2016 World Malaria Report estimating a 40% reduction in incidence of malaria between 2010 and 2015 [1]. While some have attributed this reduction to the bi-national elimination plan, few malaria control strategies have actually been implemented [7–8]. Furthermore, due to natural disasters much of the country’s public health infrastructure was incapacitated during this time period [9]. The goal of this study was to investigate the trends in the number of malaria diagnostic tests given, the number of positive results, and the test positive rate before the earthquake when microscopy was used and during the recovery period after the national adoption of malaria RDTs.

## Methods

### Study location and clinic characteristics

**Fig 1:**
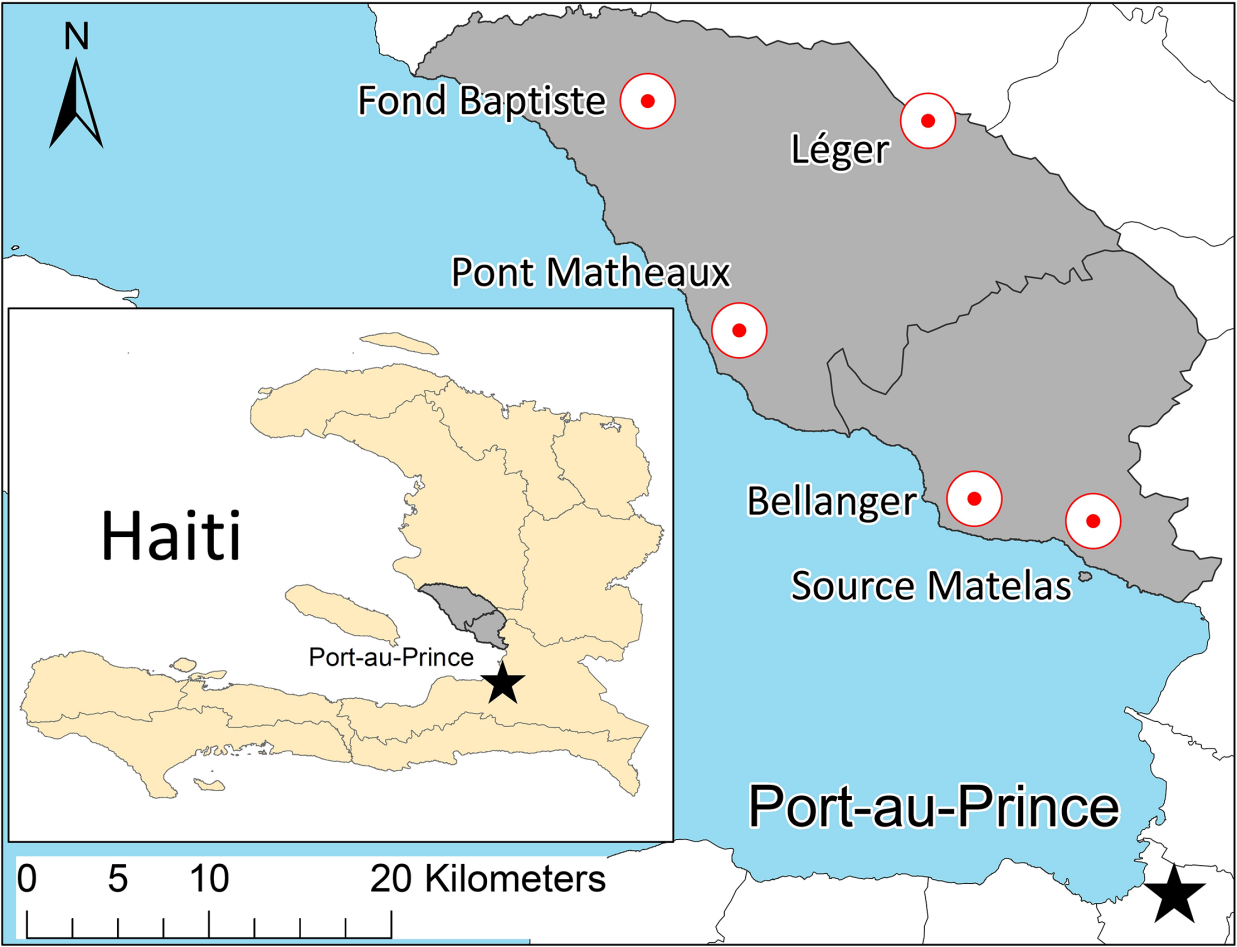
Geographical location of medical clinics from the Ouest Department of Haiti. The geographical locations of the clinics within the African Methodist Episcopal Church Service and Development Agency (AME-SADA) network are presented with respect to the national administrative boundaries (departments in inset, communes in map). These include Fond Baptiste, Leger, and Pont Matheaux from the commune of Arcahaie (estimate population 118,501); along with Bellanger and Source Matelas from the commune of Cabaret (estimated population 62,063).

Hospital records were obtained from facilities within the African Methodist Episcopal Church Service and Development Agency (AME-SADA), a non-profit network of medical clinics in the Ouest Department of Haiti headquartered in Washington, DC [10]. These included Fond Baptiste, Léger, and Pont Matheaux from the commune of Arcahaie (estimated population 118,501); along with Bellanger and Source Matelas from the commune of Cabaret (estimated population 62,063). The geographic locations of the five clinics are presented in Fig 1 with respect to the national administrative boundaries of Haiti. The clinic located in Pont Matheaux is the largest location with 9,277 patient visits per year; whereas the other four clinics are satellite locations that often serve less developed areas with a combined total of 2,298 patient visits per year. The clinical network reports statistics to the MSPP in the form of standardized monthly reports that are aggregated by the departmental headquarters for the Ouest Department (Department Sanitaire De L’Ouest) to generate national-level health statistics [11]. These data included the number of febrile patients tested for malaria and the number of malaria tests with a positive result.

#### Data extraction

Monthly records were available from the AME-SADA clinics in the form aggregated patient data between the years 2008 to 2013. After obtaining the reports, two researchers independently extracted the data into a spreadsheet using Microsoft Excel (Microsoft Corp., Redding, WA, USA). The fields extracted included the name of the clinic submitting the report, the month of the report, year of report, total number of patient visits that month, number of patients tested for malaria each month, and the number of positive malaria tests. After data entry, the spreadsheets generated by both researchers were compared for inconsistencies, which were checked against the original documents until both were in complete agreement prior to the analyses as described in the subsequent section. Rainfall data reported in inches per month between January 1^st^, 2008 to January 1^st^, 2017 were obtained from the Tropical Rainfall Measuring Mission (TRMM) for the area corresponding to the Arcahiae Arrondissement (polygon bounded by the following coordinates -72.859 E, 18.655 S, -72.178 W, 19.166 N) [12].

#### Time series data structure

**Fig 2:**
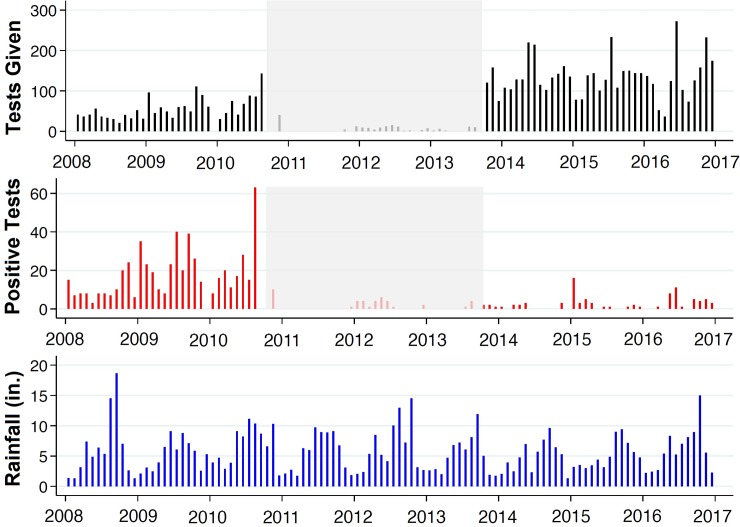
Monthly totals of malaria diagnostic tests, positive results, and rainfall. The monthly totals of malaria diagnostic tests used (black), number of positive test results (red), and rainfall in inches (blue) between January 1, 2008 to January 1, 2017 are presented. The shaded region in the top two plots represents a period of incomplete records and/or reduction of services between September 2011 and September 2013 following a period of disaster.

The resulting data are the total number of patients tested for malaria, number of positive malaria tests, and the total amount of precipitation in inches by month. As shown in Fig 2, the data can be conceptually separated into three approximately equal periods of time with complete records from January 2008 to September 2010 (months 1–32), frequently missing or incomplete reports between September 2010 and September 2013 (months 33–69), and complete data from September 2013 to January 2017 (months 70–108). During the first period of time from January 2008 to September 2010, a standardized microscopy protocol endorsed by the World Health Organization was the only method of malaria diagnosis used at the AME-SADA clinics [13]. Data from the second period of time between September 2010 and September 2013 (months 33–69) correspond to the months that followed the January 2010 earthquake, onset of the cholera outbreak in October 2010, and Hurricane Sandy in October of 2012; all of which interrupted medical services. During the third period of time from September 2013 to January 2017, AME-SADA clinics exclusively used the CareStart Malaria HRP2 rapid diagnostic test (Access Bio, Inc., Monmouth Junction, New Jersey, USA) to diagnose all suspected malaria patients.

#### Comparison of malaria diagnostic testing over time

Descriptive summaries of the number of patients tested for malaria and the number of positives tests were generated by time period for individual clinics, for small and large clinics separately, and for all clinics combined (Table 1). The number of positive results was divided by the number of febrile patients tested for malaria to calculate a test positive rate (TPR) expressed as a percentage (%). Generalized linear models (GLM) with a Poisson distribution were used to model the number of febrile patients tested for malaria, the number of positive tests, and the test positive rate (TPR). The association between the study time period and the three aformentioned outcomes was explored using only data from the clinics that tested patients before and after the adoption of RDTs (Bellanger and Pont Mattheux). First, the three time periods were coded as categorical variables representing the pre-earthquake period (coded 1), the post-disaster period (coded 2), and the post-recovery period (coded 3). The number of febrile patients tested per month was compared using the period as a categorical predictor and included all three time periods. The number of positive tests per month was compared using the time period as a categorical predictor and the number of febrile patients tested as a covariate. The TPR by month was compared using the time period as a categorical predictor and the number of febrile patients tested as an offset to express the TPR as a rate. Due to variability in the TPR during months where few patients were tested, the comparison of the the number of positive tests and the TPR included only the months where at least 20 febrile patients were tested. Since the changes in season could have influenced the TPR or increased the number of febrile patients tested, the default model was also evaluated with the inclusion of a seasonal term. Fourier terms (two pairs of sine and cosine functions) computed by dividing the time elapsed in months (*T*) by 12, multiplication by 360 degrees, and inclusion in the previous model as a third term representing the product of the sine and cosine functions with time. All model coefficients were expressed as incidence rate ratios (IRRs) with 95% confidence intervals and statistical significance denoted by an alpha value of 0.05. All statistics were conducted using STATA (StataCorp, College Station Texas, USA).

**Table 1:** Malaria diagnostics given to febrile patients and positive tests by clinic.

	Microscopy (2008 to 2011)			Rapid Test (2013 to 2017)		
Clinics	tested	positive	TPR (%)	tested	positive	TPR (%)
Bellanger	340	134	39.41	425	11	2.59
Pont Mattuex	1387	423	30.50	2866	25	0.87
*Combined*	1727	557	32.25	3291	36	1.09
Leger	0	NA	NC	31	0	0.00
Fond Baptist	0	NA	NC	124	1	0.81
Source Matelas	0	NA	NC	1686	46	2.73
*Combined*	0	NA	NC	1841	47	2.55
*Total*	1727	557	32.25	5132	83	1.62

The number of febrile patients tested for malaria, the number of positive tests, and test positive rate (TR) are presented by month for all available data. Between 2008 and 2011 microscopy was used to diagnose malaria infections; during years 2013 to 2017 diagnosis was made exclusively with rapid diagnostic tests. The data are presented for individuals clinics, separately for clinics that did not conduct both microscopy and RDTs, and all clinics combined.

## Results

### Comparison trends in malaria diagnoses before and after the adoption of RDT

In the clinical network, the number of febrile patients tested increased from 1,727 patients between January 2008 and October 2011 to 5,132 between January 2014 and September 2016. Despite an increase in testing, the number of positives decreased from 557 to 83 confirmed malaria diagnoses during the same time period, corresponding to a decrease in the TPR from 32.25% to 1.62% (Table 1). From Table 1 it is also evident that prior to adoption of RDTs, only two clinics (Pont Matheaux and Bellanger) were able to perform microscopy to diagnose malaria. The adoption of the RDT led to an increase in the number of febrile patients tested by Fond Baptiste, Léger, and Source Matelas (Table 1). Since not all clinics had the capacity to diagnose malaria before and after the adoption of RDTs, a comparison of the number of tests given and the TPR was conducted using time-series data from Pont Matheaux and Bellanger (Table 2).

**Table 2:** Malaria diagnostics given to febrile patients and positive tests by year.

			Bellanger/Pont Mattuex			
Year	months	tested	positive	TPR (%)	95% conf. int. TPR	
2008	12	449	124	27.62	18.45	37.41
2009	11	658	246	37.39	29.40	51.89
2010	7	474	136	28.69	17.31	34.85
2011	2	16	1	6.25	0.00	57.11
2012	11	81	26	32.10	11.60	38.90
2013	7	369	5	1.36	0.00	4.85
2014	12	1238	5	0.40	0.00	0.92
2015	12	864	18	2.08	0.00	2.87
2016	12	922	13	1.41	0.38	2.39

The number of febrile patients tested for malaria, the number of positive tests, and test positive rate (TPR) with 95% confidence intervals are presented by year for the two clinics (Bellanger and Pont Mattuex) conducting malaria diagnoses in all three study periods. Due to missing data, the number of months available is included in the first column; months with less than 20 tests given are included in this table. Microscopy was used exclusively to diagnose febrile patients with malaria until 2012; by 2013 both clinics had completey adopted the RDT for diagnosis of malaria.

**Fig 3:**
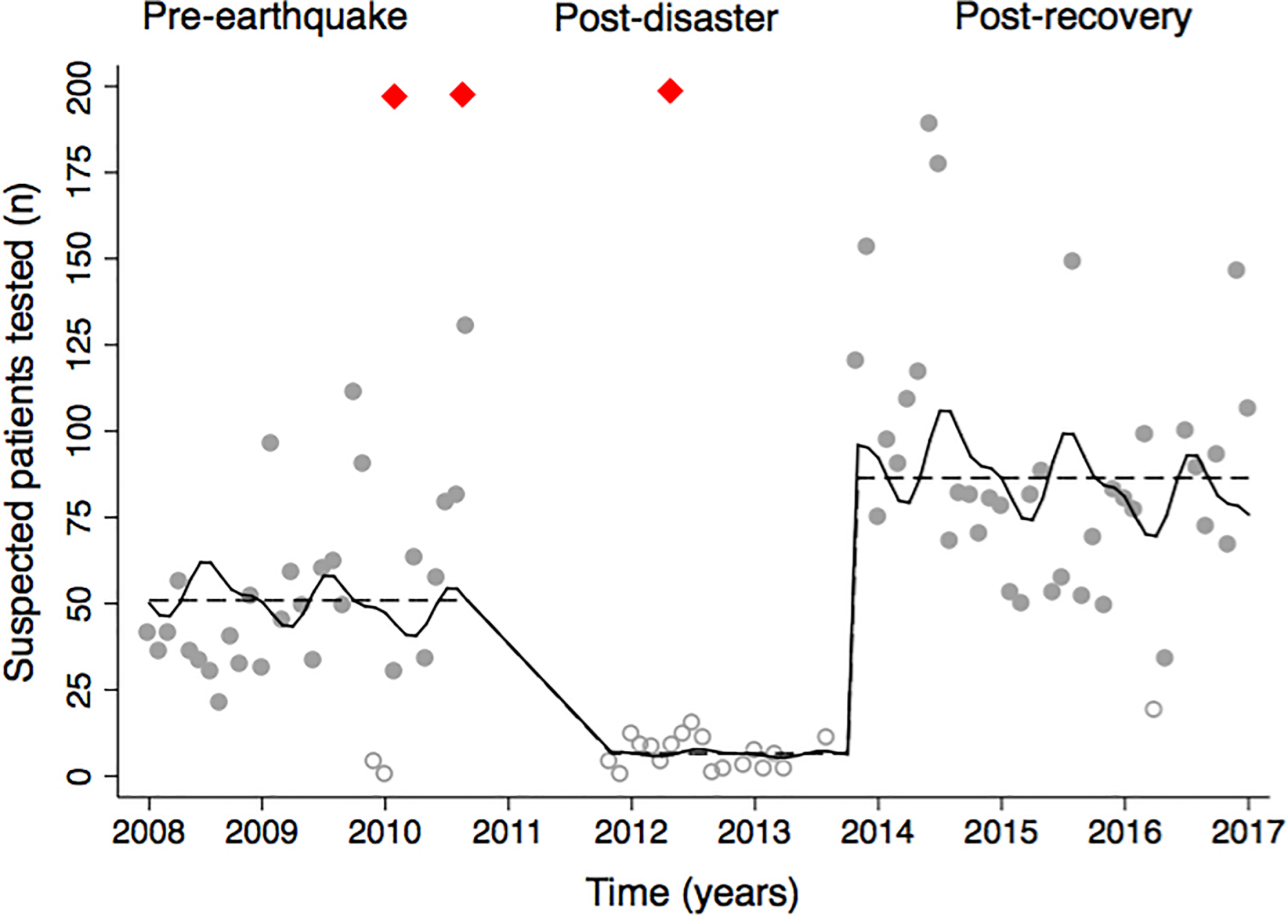
The number of suspected malaria patients tested before, during, and after the earthquake. The number of patients tested for malaria infections at Bellanger and Pont Matthuex between January 1, 2008 and January 1, 2017 are presented (gray dots) with reference to three national disasters (black diamonds) including an earthquake in January 2010, cholera outbreak in October 2010, and hurricane Sandy in Mar 2012. The number of tests were modeled as three discrete time periods: before the earthquake (January 2008 to September 2011), post-disaster (September 2011 to September 2013), and post-recovery (September 2013 to January 2017); which are shown with (solid black line) and without (dashed black line) inclusion of a seasonal component in the model. Months with less than 20 patients tested appear as hollow gray circles.

The number of febrile patients tested for malaria was significantly different (P< 0.001) between the three pre-defined time periods from January 2008 to January 2017 (Fig 3). Between January 2008 and October of 2011 the clinics tested approximately 53 (95% CI 42–63) febrile patients per month. In the post-disaster period that followed the earthquake (Jan. 2010), a national cholera outbreak (Oct. 2010) and hurricane Sandy (Oct. 2012) contributed to the reduction in healthcare capacity. Compared to the pre-earthquake period, the number of febilre patients tested for malaria decreased to less than 10 (95% CI: 5,9) per month; corresponding to an 87% reduction (IRR: 0.13; 95% CI: 0.11, 0.15) in the post-disaster period. By 2014, the clinics had recovered from the various disasters and began to test over 80 (95% CI: 74–98) patients per month between January 2014 to January 2017; correpsonding to an increase in the number of patients tested by approximately 70% (IRR: 1.69; 95% CI: 1.59, 1.79) compared to the pre-earthquake period. Adjusting for the effect of season, the differences in the number of febrile patients tested compared to the pre-earthquake season was similar, with a 84% decrease in the number of patients tested in the post-disaster period (95% CI: 0.13, 0.20), and a 2.5 fold increase in the number of patients tested during the post-recovery period (95% CI: 2.06, 3.09).

**Fig 4:**
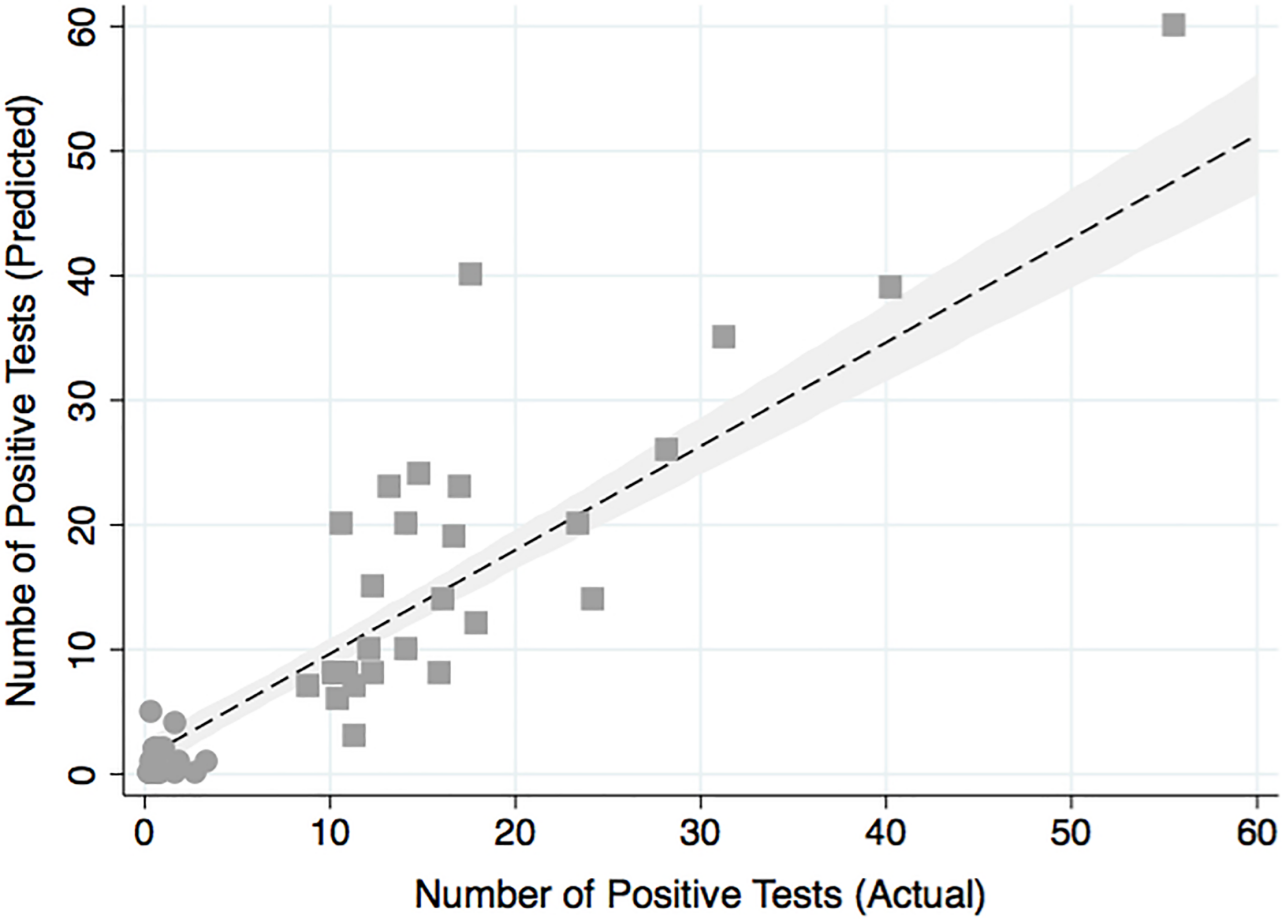
Actual and predicted number of positive tests before and after the adoption of RDTs. The actual number of positive tests and number of positive tests predicted from the regression model are shown along with a linear fit with a 95% confidence interval. Two clusters emerge, the values from period 1 (squares) when microscopy was used and the values from period 2 (circules), once RDTs were adopted. Period 2 was omitted from the regression due to large amounts of missing data or months with less than 20 febrile patients tested for malaria.

**Fig 5:**
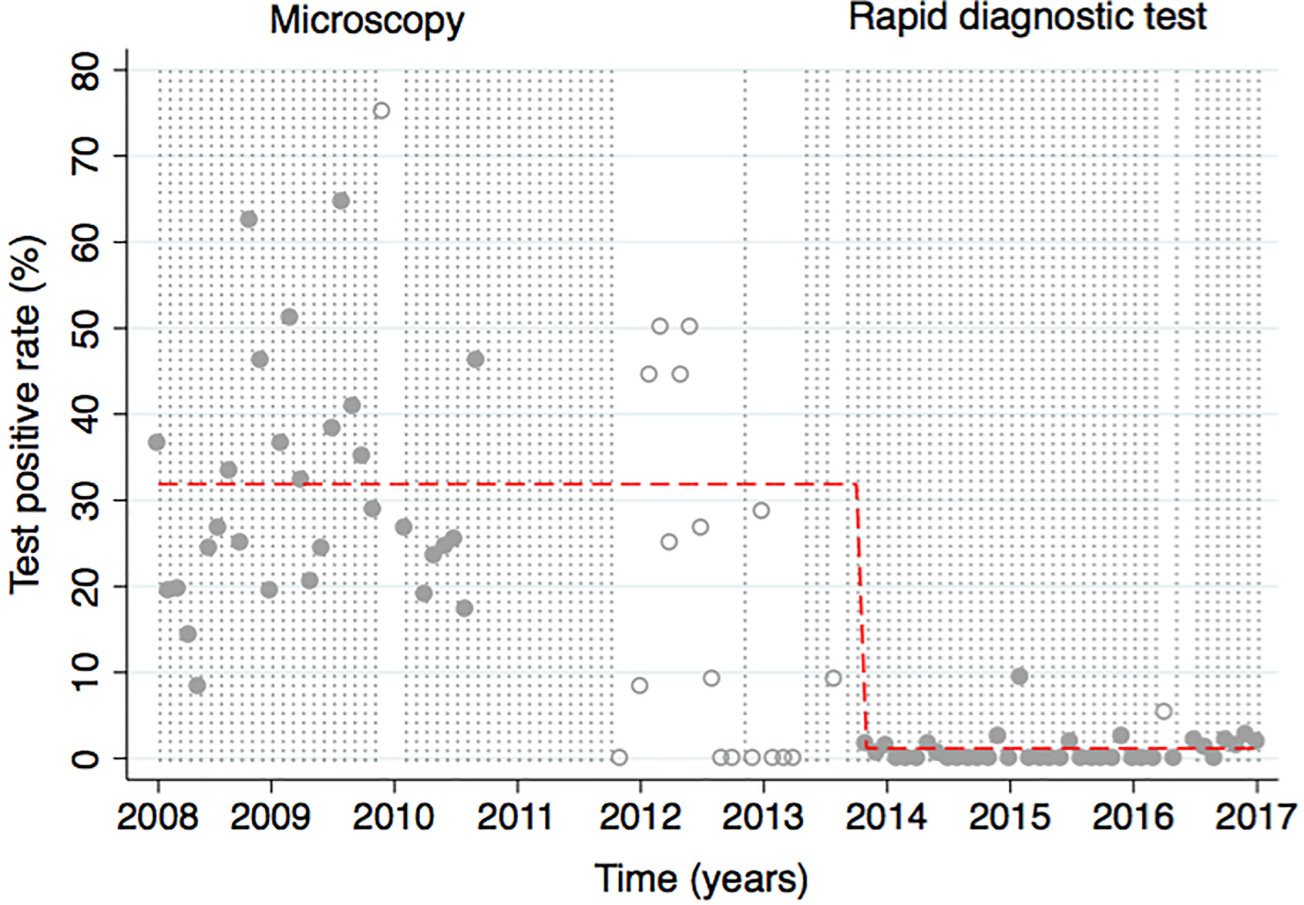
Test positive rate (TPR) before, during, and after the earthquake. The percentage (%) of diagnostic tests with a positive result (gray circles) between January 1, 2008 and January 1, 2017 are presented with the values predicted without a seasonal component included in the model (dashed red line). Months with less than 20 patients tested appear hollow gray circles with gaps in the dotted background for those months.

The number of febrile patients with a positive test result was also significantly different (P<0.001) between the three pre-defined study time periods; with an monthly average number of confirmed diagnosis of 17 people (95%CI: 12, 22) during the pre-earthquake period, 2 people (95% CI:1,3) during the post-disaster period, and 1 person (95%CI: 1, 2) in the post-recovery period. As expected, the number of positive tests was associated with the number of tests given during the same month (P< 0.001). Once included as a covariate, the model was able to predict over 85% of the variability (R^2^ = 0.85) in the number of positive tests over time (Fig 4). The plot of the actual and predicted number of positive tests also shows separation of the data into two discrete clusters corresponding to period 1 where microscopy was used and period 3 where RDTs were used. Compared to time period 1, there was a 98% reduction in the number of positive tests in period 3 (IRR 0.022; 95% CI 0.015, 0.33). This decrease in the number of positive tests and increase in the number of tests given resulted in a TPR of 32.3% (95% CI 26.5, 37.2%), 17.3% (95% CI 7.9, 26.8%), and 0.9% (95% CI 0.4, 1.57%) for time periods 1, 2, and 3, respectively (Fig 5). The TPR was variable in the post-disaster period and not significantly different (P = 0.12) than the pre-earthquake period; where 76% of months had missing data or tested less than 20 patients. Compared to the pre-earthquake period when microscopy was used exclusively, there was over a 95% decrease in the likelihood of a positive test during the post-recovery period (IRR: 0.03; 95% CI: 0.02, 0.04); when rapid diagnostic tests were used exclusively. After adjustment for the effect of season, the difference in the number of febrile patients with positive results in the post-recovery period compared to the pre-earthquake season was unchanged (IRR = 0.019; 95% CI: 0.01, 0.04).

## Discussion

Using monthly data collected before and after the adoption of malaria RDTs in Haiti, this study was able to demonstrate that the drastic reduction in malaria indicators occurred as early as September 2013. Furthermore, the rates from this sample of Haitian clinics were almost identical to those reported for the entire country in 2010 and 2015 from the 2016 World Malaria Report [1]. In 2010 the WHO reported that 270,427 microscopic diagnoses were performed in Haiti with 84,153 confirmed positive (31.1%); whereas in 2015 the number of RDTs used was 233,081 with 12,359 confirmed positive (5.3%). From the clinics offering testing before and after the adoption of the RDT (Table 1), the number of microscopic diagnoses was 1,727 with 557 confirmed positive (32.3%); whereas after the policy change, the number of RDTs used was 3,291 with 36 confirmed positive (1.09%). Though few studies have compared the ability of laboratory technicians at Haitian clinics to diagnose malaria by microscopy to trained experts, one study reported the positive predictive value (PPV) of only 22.2% [14]. Since this PPV means the local operators overestimated the actual number of patients with malaria by almost five-fold, it is plausible that the reduction in the number of reported malaria cases from 31.1% in 2010 to 5.3% in 2015 was due to the adoption of a more specific method of diagnosis.

Besides the temporary approval of three brands of RDT in 2010 and permanent adoption by 2012, other elements of the bi-national plan to eliminate malaria from Haiti must be considered in the context of this drastic decline. Concurrent with the adoption of the RDT, the recommended treatment protocol for uncomplicated malaria throughout Haiti was changed from standard chloroquine therapy (25 mg/kg total, administered over 3 days) to combined therapy with the addition of single dose of primaquine (0.25 mg/kg) [15]. This combination was intended to reduce transmission by targeting gametocytes in confirmed patients. However, only 109,625 treatment regiments of combined chloroquine/primaquine were distributed by the Global Fund in 2013; given that only 20,957 patients were confirmed to have malaria, the majority of these treatments were given empirically [16]. Between 2012 and 2013, the Global Fund also allocated approximately 3 million bed nets (ITN/LLIN) throughout Haiti free of charge to all age groups in a one-year long mass campaign [17]. The only study to investigate the impact of this one-time distribution in 2012 found that the residual permethrin content and mosquito knockdown ability met WHO-recommended functional thresholds in all Departments of Haiti, but between 6 and 30% of the nets failed (total hole area>1000 cm^2^) after 12 months and did not appear to be effective for the prevention of clinical malaria (OR = 1.00, P-value 0.971) [18]. Since the authors found no evidence of permethrin resistance in mosquitos reared from larvae collected in the five study departments, they speculated that the lack of association was due to altered feeding behavior of the primary vector, *Anopheles albimanus*, which was observed to bite outdoors or at times when people were not likely to use nets [19]. Additionally, the last phase of the plan (2013–2020) involved indoor residual spraying and active surveillance to target high-transmission foci; neither of which has been implemented. Thus, given the proportionally small allocation of effective pharmacalogical treatments in 2013, most of which were presumptively used, the lack of efficacy demonstrated by the ITN/LLN distribution in 2012, and the failure to conduct indoor spraying and active surveillance as planned, we believe it is rather unlikely that such efforts are responsible for the drastic decline in malaria incidence throughout Haiti.

Regardless of whether the decline in malaria indicators was simply due to the change in diagnostic approach or a result of elimination activities, the true incidence of clinical infections and/or prevalence of asymptomatic parasitemia in Haiti remains unclear. Even though the entire country of over 10 million people is considered at risk for malaria, with over 5 million considered high risk, the number of people living in active endemic foci also remains unknown [1]. Just as active surveillance efforts to estimate the disease burden of malaria in Haiti vary widely depending on the methods used and the location sampled, passive surveillance estimates depend on the level of clinical suspicion necessary to administer a diagnostic test and the population attending the clinic for treatment [20]. For instance, microscopic testing in suspected malaria patients can range from 0 to 14% [15], while serological testing of asymptomatic community members have estimated annual seroconversion rates between 2 and 5% [21]. Polymerase chain reaction (PCR) testing of asymptomatic community-members has estimated a prevalence of 3.1% [22], whereas the most recent study using a highly sensitive reverse-transcriptase polymerase chain reaction (RT-PCR) suggested that asymptomatic parasitemia might be present in 4 to 40% of Haitians depending on location [23]. Likewise, the ease of use to diagnose malaria with RDTs in clinics that were previously not able to perform microscopy could have led to the inclusion of a patient population that would not normally meet the level of suspicion necessary to conduct a diagnostic test; blurring the distinction between clinical management of malaria and clinic-based passive surveillance. One thing is certain, if Haiti is to succeed in malaria elimination, active surveillance to identify areas with the highest prevalence should precede interventions, which should include before and after comparisons to determine their efficacy.

One limitation of this study is that there were missing clinic records between 2010 and 2013 due to the devastation from the earthquake, cholera outbreak, and Hurricane Sandy. Thus, it is clear that the decline in the number of reported cases occurred as early as September 2013, but we were unable to determine if the reduction occurred before that time (ie. directly after the adoption of RDTs in 2012). Although the numbers from the study clinics matched the national rates of positive malaria tests when microscopy or RDTs were used in 2010 and 2015, a more extensive dataset would have increased the external validity of this study and allow for stronger inference regarding the association between this policy change and the decline in the number of reported cases. The decrease in the TPR could also be attributed to a relative increase in the number of viral illnesses such as Dengue and Chikungunya, which were present after the earthquake. However, the absolute number of positive malaria tests also drastically decreased making this scenario an unlikely reason for the decrease in TPR. Finally, as reported in other countries, it is possible that the RDTs which utilize HRP2 as the primary antigen were underdiagnosing patients infected by malaria parasites harboring HRP2 deletions [24]. The presence of HRP2 deletions in malaria parasites along with surveillance for the development of drug resistance should remain topics of future research in Haiti.

## Conclusions

The findings from this study indicate that the drastic decrease in malaria indicators occurred prior to 2015 after a national change in the method of malaria diagnosis and in the setting of minimal coordinated elimination efforts. While such elimination efforts or changes in the environment cannot be discounted, the comparison of 2010 and 2015 indicators as a measure of progress towards elimination should be made with caution as they were estimated using different diagnostic methods. As such, we recommend that future interventions proceed with before and after measurements of malaria indicators to determine their efficacy. Furthermore, active surveillance measures should be implemented on a national scale to target foci of high transmission and accurately document future progress towards malaria elimination in Haiti.

## Supporting information

S1 Data Weppelmann et al data. (XLSX)
